# OMICS Technologies and Applications in Sugar Beet

**DOI:** 10.3389/fpls.2016.00900

**Published:** 2016-06-22

**Authors:** Yongxue Zhang, Jingdong Nan, Bing Yu

**Affiliations:** ^1^Key Laboratory of Molecular Biology of Heilongjiang Province, College of Life Sciences, Heilongjiang UniversityHarbin, China; ^2^Engineering Research Center of Agricultural Microbiology Technology, Ministry of Education, Heilongjiang UniversityHarbin, China

**Keywords:** sugar beet, genomics, transcriptomics, proteomics, metabolomics

## Abstract

Sugar beet is a species of the *Chenopodiaceae* family. It is an important sugar crop that supplies approximately 35% of the sugar in the world. Sugar beet M14 line is a unique germplasm that contains genetic materials from *Beta vulgaris* L. and *Beta corolliflora* Zoss. And exhibits tolerance to salt stress. In this review, we have summarized OMICS technologies and applications in sugar beet including M14 for identification of novel genes, proteins related to biotic and abiotic stresses, apomixes and metabolites related to energy and food. An OMICS overview for the discovery of novel genes, proteins and metabolites in sugar beet has helped us understand the complex mechanisms underlying many processes such as apomixes, tolerance to biotic and abiotic stresses. The knowledge gained is valuable for improving the tolerance of sugar beet and other crops to biotic and abiotic stresses as well as for enhancing the yield of sugar beet for energy and food production.

## Introduction

**Figure 1:**
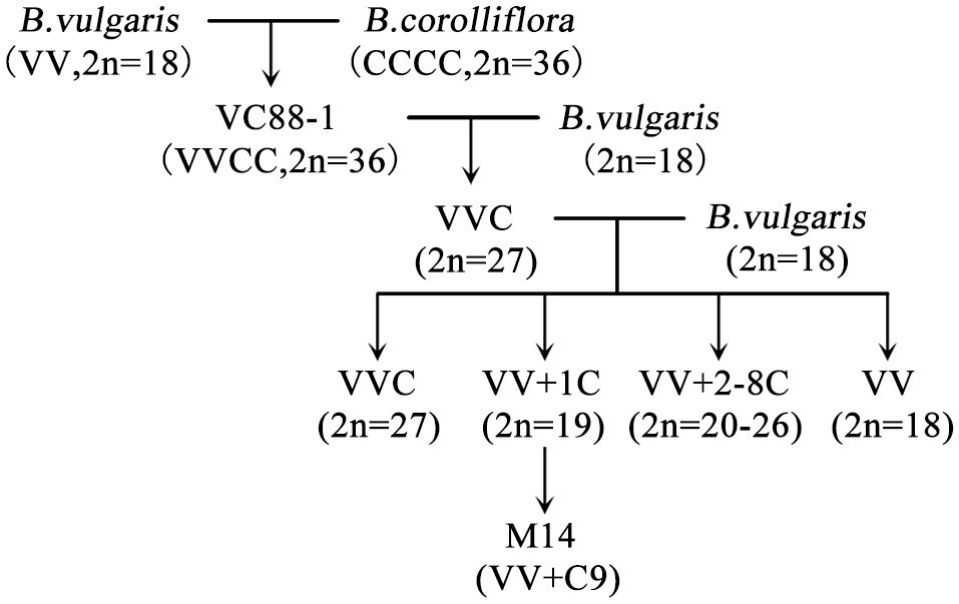
**Generation of sugar beet M14 apomict (*B. vulgaris* genome plus chromosome No. 9 from *B. corolliflora*)**.

Sugar beet (*Beta vulgaris*. L), a species of *Chenopodiaceae* family, is an important sugar crop that supplies approximately 35% of the sugar in the world (Liu et al., 2008). In the United States, sugar beet has provided about 55 percent of the total sugar produced domestically since the mid-1990s (Benoit et al., 2015). Sugar beet was introduced to China from Arabia about 1500 years ago and it is a dicotyledonous plant with high economic value in many countries. Therefore, how to grow the crop efficiently has been a priority and extensively investigated (Draycott, 2006). Sugar beet is a biennial crop which grows a sugar-rich tap root in the first year (the vegetative stage) and a flowering seed stalk in the second year (the reproductive stage; Chen et al., 2016). The types of sugar beet can be distinguished according to various internal and external features, such as economic characters, trait diversity, and chromosome ploidy. *Beta corolliflora* Zoss. (2*n* = 36) is a wild species of the beet Corollinae section that has many characteristics including tolerance to drought, cold, salt and against disease. Sugar beets (*Beta vulgaris*) are classified as salt-tolerant crops (Dunajska-Ordak et al., 2014). Scientists have studied the interspecific crossing of cultivated sugar beet (*Beta vulgaris* L. 2*n* = 19) and *B. corolliflora* Zoss. for decades (Dalke et al., 1971; Filutowicz and Dalke, 1976). In our lab, Guo et al. obtained the sugar beet monosomic addition line M14 (Figure 1), which contains the *Beta vulgaris* L. genome with the addition of No. 9 chromosome of *B. corolliflora* Zoss (Guo et al., 1994). It has several interesting characteristics including apomixes and tolerance to drought, cold and salt stress (Guo et al., 1994). Apomixis is a mode of asexual reproduction characterized by the production of clonal seeds via the parthenogenesis development of an unreduced egg. The apomictic process bypasses meiosis and egg cell fertilization, producing offspring that are exact copies of the mother plant (Nogler, 1984; Ozias-Akins, 2006). Sugar beet M14 therefore can function as a unique germplasm for studying the characteristics of apomixes and tolerance to abiotic stresses.

During evolution, plants have developed complex strategies that regulate biochemical and physiological acclimation in order to respond to biotic stress (viral, bacterial, fungal, and oomycete infections; Baum et al., 2007) and abiotic stress (salinity, drought, and low temperature; Barnabas et al., 2008; Yolcu et al., 2016). Biotic and abiotic stresses severely reduce agricultural productivity worldwide (Munns and Tester, 2008; Pinhero et al., 2011; Mishra et al., 2012). Therefore, understanding how plants respond and tolerate biotic and abiotic stresses is important for boosting plant (e.g., sugar beet) productivity under these challenging conditions. In order to minimize the negative impact of these stresses, studying how the sugar beet has evolved stress coping mechanisms will provide new insights and lead to novel strategies for improving the breeding of stress-resistant sugar beet and other crops.

In recent years, genomics knowledge based on Next Generation Sequencing (NGS), gene editing systems, gene silencing, and over-expression methods have provided a large amount of genetic information to help reveal the mechanisms of biotic and abiotic stress responses in plants (Saad et al., 2013; Shan et al., 2013; Yin et al., 2014; Luan et al., 2015). At the transcriptome level, technological innovations have made it possible to overview the changes that occur at the transcriptomic level under different environmental stress conditions. Microarrays and RNA sequencing techniques are employed to elucidate the differential expression of genes involved in biotic and abiotic stress responses in a variety of plant species (Kreps et al., 2002; Shinozaki and Yamaguchi, 2007; Ergen and Budak, 2009; Mitchell et al., 2014; Akpinar et al., 2015; Budak and Akpinar, 2015; Wang et al., 2016). Proteomics and metabolomics are two emerging “-omic” techniques in the post-genomic era (Fernandez-Garcia et al., 2011). Proteomics technologies allow the simultaneous identification and quantification of thousands of proteins that are an essential tool for understanding the biological systems and their regulations (Silva-Sanchez et al., 2015). Proteomics can be used to compare proteomes under varying stress conditions (Draycott, 2006; Liu et al., 2008; Benoit et al., 2015). Metabolomics focuses on the global profile of the low molecular weight (< 1000 Da) metabolites which are the end products of metabolisms in biofluids, tissues and even whole organism (Brosché et al., 2005). Metabolomics has recently been utilized in an increasing number of applications to investigate plant metabolite responses to abiotic stresses, particularly drought, flooding, salinity, and extreme temperatures (heat and cold; Jorge et al., 2015; Jia et al., 2016). Obviously, a combination of OMICS techniques including genomics, transcriptomics, proteomics and metabolomics could could serve to validate and complement one another in order to provide an efficient way capable of improving stress tolerance in plants.

Sugar beet is a good plant resource to explore and identify genes and proteins involved in stress resistance. Sugar beet is widely used in sugar industry (Liu et al., 2008). It is a source of the clean energy via hydrogen gas and bioethanol (Dhar et al., 2015). It contains abundant betaine and betalain metabolites. Betaine is used to improve the plant stress tolerance (Catusse et al., 2008). Betalains are natural pigments which have potential health benefits (anticarcinogenic and antioxidative) and have attracted both scientific and economic interest (Stintzing and Carle, 2007; Moreno et al., 2008). A rich and cheap source of betalains in red beet root (*Beta vulgaris* L.) is very attractive to the pharmaceutical and food industries (Wybraniec, 2005; Wybraniec et al., 2011, 2013). In this review, we have summarized OMICS applications and covered the recent discoveries in sugar beet research including the M14 for identification of novel genes and proteins related to biotic and abiotic stresses, apomixes, and metabolites related to energy and food production. The knowledge gained is valuable for improving sugar beet and other crops tolerance to biotic and abiotic stresses as well as for enhancing the yield of sugar beet for energy and food production.

## OMICS overview for discovering novel genes, proteins and metabolites in sugar beet

**Figure 2:**
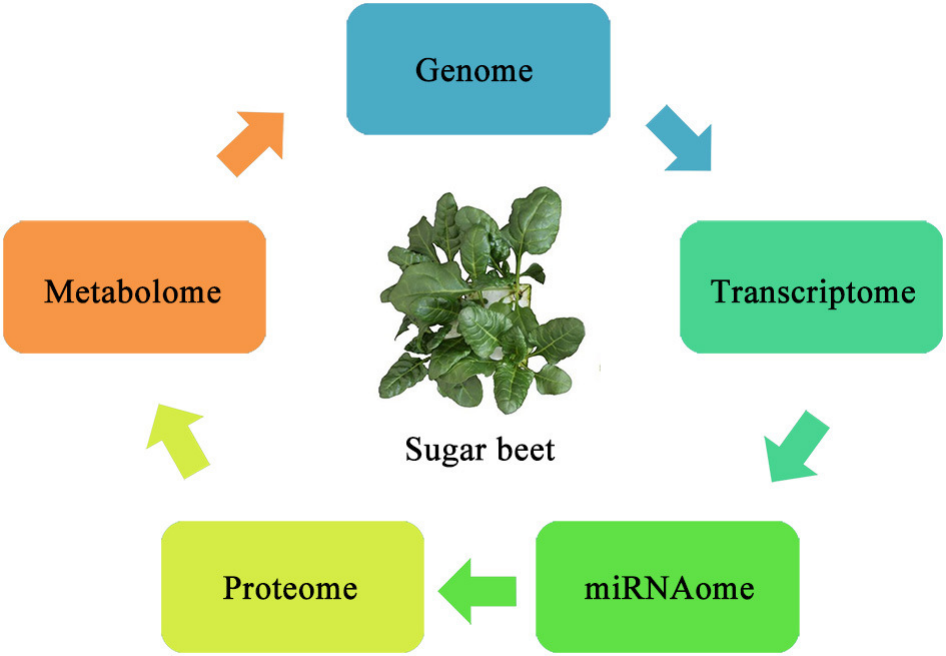
**Schematic diagram showing information flow for OMICS studies in sugar beet**.

In recent years, the use of OMICS tools has considerably increased for studying biotic and abiotic stresses in plants. The existing methods include genomics, transcriptomics, proteomics, metabolomics, and several others capable of discovering and characterizing the expression of genes or proteins during biotic and abiotic stresses with high efficiency shown in Figure 2. These highly sensitive tools can analyze plant tissues and help to improve our understanding of the tolerance mechanisms utilized by sugar beet.

## Genomics studies in sugar beet

### The whole genome sequence of sugar beet

The whole genome sequence of sugar beet has been reported by Dohm et al. (2014). A total of 27,421 protein-coding genes were predicted based on transcription data and annotated on the basis of sequence homology (Dohm et al., 2014). Compared to other flowering plants with the genome information, the sugar beet has a small number of genes encoding transcription factors. It has been suggested that the sugar beet may contain unknown genes associated with transcriptional control, and that the genetic interaction network of sugar beet may have evolved in unique ways compared with other species. Using the sugar beet genome sequence and related resources, we expected to find the molecular mechanisms underlying gene regulation and gene environment interaction. In addition, this information can help to develop crops with improved sugar and natural substance production and have an important role in future plant genomic research.

### SMRT of the sugar beet chloroplast genome

SMRT (Single Molecule Real-Time) is a third generation sequencing method, which offers much longer read length compared to NGS methods. It is well suited for *de novo*- or re-sequencing projects. It not only contains reads originating from the nuclear genome, but also lots of reads from the organelles of the target organism (Sanger et al., 1977; Liu et al., 2012). Stadermann et al. described a workflow for *de novo* assembly of the sugar beet chloroplast genome based on data originating from a SMRT sequencing dataset targeted on nuclear genome (Stadermann et al., 2015). They identified a total of 114 individual genes. Of these, 79 genes encode mRNA (i.e., proteins), 7 encode rRNA and 28 are tRNAs. Nine genes are located within the inverted repeat (IR) regions which encode 5 mRNAs, 1 rRNA, and 3 tRNAs. In comparison to the Illumina assembly, the annotation showed some differences due to changes in the underlying sequences.

### miRNAs involved in tolerance to abiotic stresses

miRNAs are small 19–23 nucleotides short non-coding RNAs, which play regulatory roles in many processes (Budak et al., 2015). miRNAs can act both at the transcriptional or post-transcriptional levels. miRNA mediated gene-silencing mechanism regulates the expression of transcription factors, phytohormones, and other developmental signaling pathways (Llave et al., 2002; Dalmay, 2006; Sunkar et al., 2007).

Earlier studies have shown that miRNAs mainly target transcription factors, but recent studies have revealed that miRNAs also target other development/stress signaling pathways, which are involved in various physiological processes, including root growth and development, response to stress, signal transduction, leaf morphogenesis, plant defenses, and biogenesis of sRNA (Curaba et al., 2014). Li J. L. et al. (2015) reported 13 mature miRNAs from 12 families using an *in silico* approach based on 29,857 expressed sequence tags and 279,223 genome survey sequences in *B. vulgaris*. The psRNA target server predicted 25 target genes for the 13 miRNAs. The target genes shown in Table 1 appeared to encode transcription factors or were involved in metabolism, signal transduction, stress response, growth, and development. However, there were no targets predicted from the current database of sugar beet for *Bvu-miR4, Bvu-miR9, Bvu-miR10, Bvu-miR11*, and *Bvu-miR12*.

**Table 1:** **Predicted functions of sugar beet miRNAs in plant development, signaling and stress responses**.

	**Target protein name**	**GO annotation (Molecular Function)**	**Function**	**References**
Bvu-miR1	Protein ARABIDILLO	Protein binding	Root development	Moody et al., 2012
Bvu-miR2	ATPase, V0 complex, c subunit	Hydrolase activity, acting on acid anhydrides, catalyzing transmembrane movement of substances	Salt stress	Lehr et al., 1999
		Proton-transporting ATPase activity, rotational me	–	–
	ATPase, V0 complex, d subunit	Hydrogen ion transmembrane transporter activity	Ion relate	Lake et al., 2004
	RanBPM-like	Protein binding	–	–
	Abscisic acid insensitive 5	Sequence-specific DNA binding transcription factor activity	Signaling	Liu and Stone, 2013
Bvu-miR3	Nucleoside phosphatase GDA1/CD39	Hydrolase activity	–	–
Bvu-miR5	Copa-prov protein	–	–	–
Bvu-miR6	Diphenol oxidase	Copper ion binding	–	–
		Oxidoreductase activity	–	–
		hydroquinone:oxygen oxidoreductase activity	–	–
	PHYA4 photoreceptor	–	–	–
Bvu-miR7	Respiratory burst oxidase-like protein	Peroxidase activity	Biotic interactions, abiotic stress and development.	Torres and Dangl, 2005
		Calcium ion binding		
		Oxidoreductase activity		
		Oxidoreductase activity, acting on NAD(P)H, oxygen as acceptor		
Bvu-miR8	Histone acetyltransferase GCN5	Protein binding	Transcription, Signaling	Benhamed et al., 2008
		N-acetyltransferase activity		
	Ethylene transcription factor	DNA binding	–	–
		Sequence-specific DNA binding transcription factor activity	–	–
	MYB6	DNA binding	Transcription, Signaling	Chang et al., 2013
		Chromatin binding		
	Nucleosome assembly protein 1-like protein 2	–	–	–
	MCM protein-like protein	ATP binding	–	–
		DNA binding	–	–
	WAVE complex SCAR2	Actin binding	–	–
	Dehydration responsive element-binding protein 1	DNA binding	Transcription, Signaling	Sun et al., 2008
		Sequence-specific DNA binding transcription factor activity		
Bvu-miR13	Transcription factor WD-repeat protein 1	Protein binding	Stress	Li et al., 2014
	Tetrapyrrole biosynthesis	–	–	–
	Hydroxymethylbilane synthase	Hydroxymethylbilane synthase activity	–	–
	Glycerol-3-phosphate O-acyltransferase	–	–	–
	Chloroplast	Glycerol-3-phosphate O-acyltransferase activity	–	–
		Transferase activity, transferring acyl groups	–	–
	Sodium/sulphate symporter	Transporter activity	–	–
	Cucumber peeling cupredoxin	Copper ion binding	–	–
		Electron carrier activity	–	–
	Elongation factor 1	Translation elongation factor activity	–	–
	Leucine-rich repeat-containing protein	Protein binding	Signal transduction, Stress response	Gou et al., 2010
	Magnesium-protoporphyrin IX monomethyl	–	–	–
	Ester [oxidative] cyclase	Oxidoreductase activity	–	–
		Metal ion binding	–	–
		Magnesium-protoporphyrin IX monomethyl ester (oxidative) cyclase activity	–	–

Several miRNAs identified have been shown to have critical roles in plants. For example, the expression of *Bvu-miR1* (Protein ARABIDILLO) in *A. thaliana* regulates multicellular root development (Moody et al., 2012). *Bvu-miR2* regulates the expression of ATPase during plant development and coordinates its induction in response to high salinity (Lehr et al., 1999). Through transcriptional regulation, it also affects the ATPase activity of magnesium chelatase subunit I in Barley (Lake et al., 2004) and abscisic acid insensitive 5 required to delay growth of germinated seedlings under environmental stress (Liu and Stone, 2013). *Bvu-miR7* targets the respiratory burst oxidase gene family, which encodes the key enzymatic subunit of the plant NADPH oxidase (Torres and Dangl, 2005). *Bvu-miR8* activates the transcription of a histone acetyltransferase GCN5 in *A. thalianaina* (Benhamed et al., 2008). Another target protein MYB6 acts as an immediate and positive activation signaling component of the active state of MLA immune receptors during transcriptional reprogramming for defense responses (Chang et al., 2013). Dehydration-responsive element-binding proteins form a major AP2/ethylene- responsive element-binding protein family and play crucial roles in the regulation of abiotic stress responses (Sun et al., 2008). *Bvu-miR13* targets WD-repeat proteins in this diverse family of regulatory proteins. To date, genome-wide characterization of this family has only been conducted in *Arabidopsis* and little is known about WD-repeat protein-coding genes in other species. Recently, it has become known that the WD-repeat protein plays an important role in cucumber stress resistance (Li et al., 2014). Other targets, e.g., leucine-rich repeat proteins, plays critical roles in both animal and plant signaling pathways regulating growth, development, differentiation, cell death, and pathogenic defense responses (Gou et al., 2010). These studies have provided insights into the molecular mechanisms of the miRNAs and may have great potential for sugar beet improvement. The functions of these interesting miRNAs in sugar beet need to be investigated in the future.

### QTL mapping for disease resistance in sugar beet

Leaf spot is one of the most serious and widespread foliar diseases of sugar beet. It causes necrotic lesions and progressive destruction of the plant's foliar structure and function (Holtschulte, 2000). The disease has greatly impacted on the yield and sugar contents of the crop. Doubled haploids (DHs), F2 populations of recombinant inbred lines (RILS), and near isogenic lines (NILS) are suitable populations for quantitative trait loci (QTL) mapping (Ibrahim et al., 2012). In order to deal with the complex inheritance of resistance to *Cercospora* leaf spot (CLS), Taguchi et al. (2011) used RILs of sugar beet, which were generated by a cross between a resistant line (“NK-310mm-O”) and a susceptible (“NK-184mm-O”) line. These RILs were then tested for their resistance to the CLS pathogen in the field (Taguchi et al., 2011). Composite interval mapping (CIM) showed four QTLs involved in CLS resistance that were consistently detected. There were two resistant QTLs (qcr1 on chromosome III, qcr4 on chromosome IX) that promoted resistance in the cross between lines (“NK-310mm-O”). There were two further QTLs (qcr2 on chromosome IV, qcr3 on chromosome VI) which promoted resistance in the susceptible line. In addition, a number of important resistance gene cluster have been mapped on the chromosome III in the sugar beet genome, for example: a CLS resistance QTL (Setiawan et al., 2000), the genes Rz1 toRz5 (Grimmer et al., 2007), gene Acr1 (Taguchi et al., 2010), gene RGAs (Lein et al., 2007), and gene X, a restorer of fertility for Owen CMS (Hagihara et al., 2005). These resistance-gene clusters on the chromosome III are mainly responsible for the disease resistance in sugar beet.

### BAC library from the genomic DNA of the No. 9 chromosome in sugar beet M14

A plant-transformation-competent binary BAC library was constructed from the genomic DNA of the No. 9 chromosome in sugar beet M14 (Fang et al., 2004). A total of 2365 positive clones were obtained and arrayed into a sublibrary specific for the *B. corolliflora* chromosome 9 (designated bcBAC-IX). The bcBAC-IX sublibrary was further screened with a subtractive cDNA pool generated from the ovules of M14 and the floral buds of *B. vulgaris* by the suppression subtractive hybridization (SSH) method. One hundred and three positive binary BACs were obtained, which may potentially contain the genes of the alien No. 9 chromosome that is specifically expressed during the ovule and embryo development of M14, and which may be associated with apomictic reproduction. The binary BAC clones are useful for the identification of the genes responsible for apomixes by genetic transformation.

## Transcriptomics studies in sugar beet

### Suppression subtractive hybridization (SSH) applications in sugar beet

SSH is a technique used to identify differentially expressed genes in cells important for growth and differentiation (Lukyanov et al., 2007). This method has often been used to study molecular mechanisms of plants in biotic and abiotic stresses (Sahebi et al., 2015). The response to insect pests in the root of sugar beet is an interesting area of plant defense research.

Puthoff et al have identified more than 150 sugar beet root ESTs enriched for genes that respond to feeding by the sugar beet root maggot in both the moderately resistant genotype F1016 and the susceptible F1010 using SSH [49]. The differential expression of the root ESTs was confirmed via RT-PCR. The ESTs were further characterized using microarray-generated expression profiles from the F1016 sugar beet roots following mechanical wounding and treatment with the signaling molecules methyl jasmonate, salicylic acid and ethylene. Of the examined ESTs, 20% were regulated by methyl jasmonate, 17% by salicylic acid and 11% by ethylene, suggesting that these signaling pathways are involved in sugar beet root defense response. Identification of these sugar beet root ESTs provides knowledge concerning plant root defense and will likely lead to the development of novel strategies for the control of the sugar beet root maggot (Puthoff and Smigocki, 2007).

SSH was applied to isolating taproot expressed genes from sugar beet as well (Kloos et al., 2002). The taproot of sugar beet (*Beta vulgaris* L.) undergoes a specific developmental transition in order for it to function as a storage organ. SSH was utilized to isolate cDNA fragments of genes expressed in the taproot. Molecular analysis of six cDNAs that encoded complete gene products revealed that these genes comprise homologs of a drought-inducible linker histone, a major latex-like protein, a phosphoenolpyruvate carboxylase kinase, a putative vacuolar processing enzyme, a thaumatin-like protein and an alanine- and glutamic acid-rich protein. All of these genes are transcribed in taproots, while the expression in leaves is low or undetectable. SSH had also been used in the sugar beet M14 to identify differentially expressed genes. A subtractive cDNA library was prepared by SSH between the flower organ of M14 and that of *B. vulgaris* (Ma et al., 2011). A total of 190 unique sequences were identified in the library and their putative functions were analyzed using Gene Ontology (GO). All of the ESTs provide information about candidate genes useful for studying M14 reproductive development. One of the genes, designated as *BvM14-MADS box*, encodes a MADS box transcription factor. It was cloned from M14 and over-expressed in transgenic tobacco plants. Overexpression of *BvM14-MADS box* led to significant phenotypic changes in tobacco (Ma et al., 2011).

Li et al. (2009) reported a comparative proteomic and transcriptomic study of the sexual and apomictic processes in sugar beet. The cDNA libraries were constructed using SSH with the apomictic monosomic addition line M14 as the tester and *B. vulgaris* as the driver. Comparative analyses of proteomic data and transcriptomic data showed that eight proteins had significant agreement between protein and mRNA expression levels. Most of the matched proteins were associated with metabolism. Interestingly, two of the matched proteins, cystatin, and thioredoxin peroxidase, were found to be associated with disease and defense response, indicating that defense-related proteins may participate in the apomictic reproductive process. Yang et al. (2012) reported transcriptomic analysis of sugar beet M14 leaves and roots that were treated with 500 mM NaCl for 7 days. The SSH technology was used to produce a high quality subtractive cDNA library. A total of 600 positive clones were randomly selected and subjected to DNA sequencing, and 499 non-redundant ESTs were obtained. After assembly, 58 unigenes including 14 singletons and 44 contigs were obtained. Some salt-responsive genes were identified as important in metabolism (e.g., sadenosylmethionine synthase 2 (SAMS2) and nitrite reductase), photosynthesis (e.g., chloroplastic chlorophyll a–b binding protein 8), energy (e.g., phosphoglycerate kinase), protein synthesis (e.g., 60S ribosomal protein L19-3), and degradation (e.g., cysteine protease and carboxyl-terminal-processing protease), and stress and defense [e.g., glutathione S-transferase (GST)]. This study has revealed candidate genes for detailed functional characterization, and has set the stage for further investigation of salt tolerance mechanisms in sugar beet.

### Transcriptomics of sugar beet in response to low temperature stress

Comparative transcriptomics is used to identify differences in transcript abundance between different cultivars, organs, development stages and/or treatment conditions (Mardis, 2008; Schuster, 2008; Bräutigam and Gowik, 2010). Low-temperature stress is a significant factor effecting of crop quality and causing production losses in agriculture. The survival of young sugar beet seedlings and the subsequent sugar yield of mature plants are often seriously limited by low temperature, especially when the plants are exposed to freezing temperatures at early developmental stages. Moliterni et al. (2015) determined the transcriptomic changes using high-throughput sequencing of the leaves and root RNAs (RNA-Seq) from sugar beets which had been exposed to cold stress which mimicked the conditions of spring nights sometimes experienced by young seedlings (Moliterni et al., 2015). In the root tissue, *CBF3* is up-regulated within a few minutes of cold stress. The authors suggested that *CBF3* transcription in the stressed plants is either maintained for a longer period or begins earlier in roots compared to leaves. The *AP2/ERF* family genes were also found to be either activated or up-regulated in all the organs by cold stress. This is an expected result, as it is known that these TFs are rapidly induced upon exposure to low temperature in *Arabidopsis* (Lee et al., 2005). *AP2/ERF* TFs are involved in the regulation of primary and specialized metabolism and in a number of JA responses (Licausi et al., 2013). It has been reported that the lack of *ADA2b* TFs leads to an increase of freezing tolerance by affecting nucleosome occupancy in *Arabidopsis* (Vlachonasios et al., 2003). In addition, a putative histone acetylase and a lysine-specific demethylase are strongly up-regulated in the leaves under cold stress, implicating chromatin remodeling, and modification in the response.

These studies suggested that the metabolic pathway most affected by low temperature was carbohydrate metabolism. In addition, the authors found 13 differentially expressed sequences related to phospholipid secondary metabolism, none of which were common to leaves and roots, implicating this pathways as another important component in early cold signaling in sugar beet roots. The high degree of organ specificity is probably due to the repertoire of compounds synthesized by the two organs upon stress. This data has illuminated the transcriptome of young sugar beet during cold stress at night, and has detailed both organ-specificity and shared pathways in the physiological response to low temperatures. These RNA-Seq based transcriptomics techniques are an effective and powerful tool, with the analyses identifying novel genes for future studies.

## Proteomics studies in sugar beet

Proteomic analysis has been carried out to address several important questions in many processes, such as: signaling, regulatory processes, and transport in plants (Zhang et al., 2008). Important knowledge of the proteomic response to stress has been mainly derived from studies of the model plants *A. thaliana* and rice (Janmohammadi et al., 2015; Liu et al., 2015; Xu et al., 2015). Proteomic analysis provides an important way to test the changes in protein levels to help identify novel proteins.

### Proteins in different sugar beets without stress treatment

Zhu et al. (2009) compared the proteomes of the monosomic addition line M14 and *B. vulgaris* using 2-DE (two-dimensional gel electrophoresis). They have identified 27 protein spots using MALDI-TOF MS. Among them, only two protein spots were found in *B. vulgaris* and five protein spots were unique to M14. These proteins were involved in many biological pathways. The results may be useful for us to better understand how genotype differences relate to proteome and phenotype differences.

Li et al. (2009) reported a comparative proteomic and transcriptomic analysis of the sexual and apomictic processes in sugar beet. A total of 71 differentially expressed protein spots from the floral organs of the M14 were identified in the course of apomictic reproductive development using 2-DE and MS analysis. The differentially expressed proteins were involved in several processes which may work cooperatively to promote apomictic reproduction, generating new potential protein markers important for apomictic development.

### Proteome changes in response to salt stress of sugar beet

To date, some proteomic studies concerning the response of sugar beet to salt stress have been reported. Wakeel et al. identified six proteins from sugar beet shoots and three proteins from roots that significantly changed under 125 mM salt treatment (Wakeel et al., 2011). Our group has performed proteomic analysis of the monosomic addition line M14 under 500 mM salt stress for 7 days. A total of 71 differentially expressed protein 2D spots were identified using LC-MS/MS. The largest functional group is represented by metabolism (28%), followed by energy (21%), protein synthesis (10%), stress and defense (10%), destination proteins (8%), unknown proteins (8%), secondary metabolism (5%), signal transduction (4%), transporters (1%), and cell division (1%). Of the identified proteins, only eight had corresponding transcriptomic data. This highlights the importance of expression profiling at the protein level (Li et al., 2009). On this basis, we focused on the functions of cystatin (Wang et al., 2012), glyoxalase I (Wu et al., 2013), CCoAOMT, and thioredoxin peroxidase. All of these proteins showed increased protein levels under salt stress. Transgenic plants exhibited enhanced tolerance to salt stress. This research has directly improved our understanding of mechanisms underlying the M14's high salt tolerance.

Another proteomics study aims to identify salt-responsive proteins in the M14 plants under 0, 200, and 400 mM NaCl mild salt stress conditions using 2D-DIGE to separate the proteins from control and salt-treated M14 leaves and roots (Yang et al., 2013). The differentially expressed proteins were identified using nanoflow liquid chromatography (LC)−MS/MS and Mascot database searching. As a complementary approach, iTRAQ LC−MS/MS was employed to identify and quantify differentially expressed proteins during salinity response in M14. We have identified 86 protein spots representing 67 unique proteins in leaves, and 22 protein spots representing 22 unique proteins in roots. In addition, 75 differentially expressed proteins were identified in leaves and 43 differentially expressed proteins were identified in roots, respectively. The proteins were mainly involved in photosynthesis, energy, metabolism, protein folding and degradation, and stress and defense. Compared to the transcriptomic data, 13 proteins in leaves and 12 proteins in roots showed significant correlation in gene expression and protein levels. These results suggest that there are several processes underlying the M14 tolerance to salt stress.

Our group also reported the changes in membrane proteome of the M14 plants in response to salt stress (0, 200, 400 mM NaCl; Li H. et al., 2015). We have used an iTRAQ two-dimensional LC–MS/MS technology for quantitative proteomic analysis. In total, 274 proteins were identified and mostly of them were membrane proteins. A total of 50 differential proteins were identified, with 40 proteins showing increased expression and 10 with decreased expression. The proteins were mainly involved in transport (17%), metabolism (16%), protein synthesis (15%), photosynthesis (13%), protein folding and degradation (9%), signal transduction (6%), stress and defense (6%), energy (6%), and cell structure (2%). These results have revealed that membrane proteins contribute to the salt stress tolerance observed in M14.

### Proteome changes in response to drought stress of sugar beet

Hajheidari et al. (2005) studied the proteome changes of sugar beet in response to drought stress. Leaves from well-watered and drought treated plants at 157 days after sowing were collected. The changes of proteins were analyzed using 2D-DIGE followed by image analysis. More than 500 protein spots were detected, and 79 spots had significant changes under drought stress. Twenty protein spots were digested and subjected to LC-MS/MS, and 11 proteins involved in oxidative stress, signal transduction and redox regulation were identified. These proteins may be important targets for improving plant abiotic stress tolerance via breeding.

## Metabolomic studies in sugar beet

Metabolomics is an exciting technology, which was used to identify secondary metabolites important for physiological processes and different stress responses (Capuano et al., 2013). During plant development and interaction with the environment, the dynamic metabolome reflects the plant's physiological and biochemical processes, and can determines the phenotypes and traits (Fernie et al., 2004; Oksman-Caldentay and Saito, 2005). Now there are gas chromatography (GC)–MS, LC–MS, capillary electrophoresis (CE)–MS, and nuclear magnetic resonance (NMR) as the major analytical tools in metabolomics. Kazimierczak et al. (2014) determined the levels of metabolites in both raw beet root and naturally fermented beet root juices from organic (ORG) vs. conventional (CONV) products. The aim of the paper was to find out the value of the fermented beetroot juices in terms of anticancer properties. The results showed that ORG fresh beetroots contained more useful compounds than CONV beetroots, such as dry matter and vitamin C, more than CONV beetroots. Compared to the CONV juice, it was found that the ORG fermented juices have stronger anticancer activity. Metabolomics is still in its infancy with these analyses of sugar beet being rare, but future research can be expected to implement this powerful technology.

## Important applications of sugar beet to be enhanced by OMICS

Many plants accumulate glycine betaine (betaine) to regulate biochemical and physiological processes under abiotic stresses (Takabe et al., 2006). For example, glycine betaine serves as a methyl donor in several biochemical pathways (Pummer et al., 2000). Sugar beet is a betaine-accumulating dicotyledonous plant with high economic value (Catusse et al., 2008). It has been reported that betaine is synthesized by the two-step oxidation of choline in which choline monooxygenase (CMO) catalyzes the first step, and betaine aldehyde dehydrogenase (BADH) performs the second step (Yamada et al., 2009). CMO is therefore a key enzyme to protect plants against abiotic stresses. It has been found in Chenopodiaceae and Amaranthaceae, but not in some betaine-accumulating plants such as mangrove (Bhuiyan et al., 2007). Unlike sugar beet, many plants do not have the betaine biosysthesis pathway. Therefore, genetic engineering of the betaine biosynthesis pathways represents a potential way to improve the plant stress tolerance (Hibino et al., 2001; Fitzgerald et al., 2009).

In addition to betaine, betalains are rich in red beets and exist only in 10 families of plants of the *Caryophyllale*. Red beetroot (*B. vulgaris*) is widely used as a food ingredient because of its beetroot red color. Therefore, most studies on red beetroot constituents have focused on the betalains. Currently, the yield of betalains extracted and purified from the beetroot red is only about 10%. Phenolics (such as betalains) have been shown to have nutritional value and there has been an increasing interest utilizing these plant constituents to improve food ingredients and as antioxidants. Betalains are important plant phenolics with many attractive properties, such as: stability, antioxidant activity, antitumor properties, and reduction of blood lipid and sugar levels. In addition, betalains are effective free-radical scavengers, which help to maintain health and protect from diseases such as cancer and coronary heart disease (Kujala et al., 2000; Han et al., 2015; Mikołajczyk-Bator et al., 2016).

Additonally, sugar beet provides approximately 30% of the world's annual sugar production and is a source of both bioethanol and animal feed. Dhar et al. (2015) have developed two highly efficient methods to produce hydrogen gas from sugar beet juice as a clean energy source (Dhar et al., 2015). Sugar beet byproducts (SBB) generated during industrial sugar extraction are mainly composed of pulp and molasses and the use of SBB as a renewable energy resource could add additional economic and environmental benefits (Aboudi et al., 2015).

OMICS research can greatly enhance potential applications of sugar beet in at least three ways. One is to improve our knowledge of molecular networks involving key metabolite synthesis, e.g., glyceine betaine and betalains. The knowledge will enable modeling and rationale engineering of the important metabolites. Additionally, we can utilize OMICS to investigate the global molecular changes that occur in response to stress and the tolerance of sugar beet to stress conditions. This information can help to improve stress tolerance and thereby yields of sugar beet, even under non-ideal conditions. Finally, research on unique sugar beet germplasms (e.g., M14 under salt stress) may be useful for enhancing yield, and food and bioenergy production in other crops.

## Conclusion

In this review, we have summarized OMICS technologies and applications in sugar beet including: M14 for identification of novel genes, proteins related to biotic and abiotic stresses, apomixes, and metabolites related to energy, food and human health. Genomics is a powerful technology to provide the whole genome blueprint of sugar beet. Mechanisms underlying apomixes and stress tolerance have mainly been studied using transcriptomics and proteomics technologies, while metabolomics studies in sugar beet are still rare. To date, a lot of genes and proteins related to apomixes and salt stress were identified to reveal apomixes and salt tolerance mechanisms in a special germplasm sugar beet M14. The results have enhanced our understanding of the molecular mechanisms of sugar beet in response to tolerance to biotic and abiotic stresses and apomixes, which may be applied to improving stress tolerance of sugar beet and other crops to improve food production, energy output (e.g., hydrogen gas and bioethanol), and accumulation of health promoting chemicals (such as betalains). Despite the use of sugar beet to produce the clean energy of hydrogen gas and bioethanol and to isolate betalains used for natural food colorants, dietary supplements and medicines were not widely applied in market, sugar beet as a high economic value crop will have a prosperous perspective of application in the food, bioenergy and pharmacy industries.

## Author contributions

YZ collected and analyzed references for this paper, and wrote the first draft, JN drew the figure and assisted in the reference organization. BY played a supervision role, and led the writing and organization. All three authors have edited the manuscript.

### Conflict of interest statement

The authors declare that the research was conducted in the absence of any commercial or financial relationships that could be construed as a potential conflict of interest.
